# “Clinical café meeting” - a clinician peer support and case discussion meeting: A tool for reflective practice and consolidation of resilience

**DOI:** 10.1192/j.eurpsy.2023.2181

**Published:** 2023-07-19

**Authors:** A. Raji, J. Rogers, B. Foley, B. Roxburgh, S. Nawaz, S. Milligan, J. Ali, Z. Shullaih, Y. Libbus

**Affiliations:** 1 Psychiatry, Dalhousie University, Halifax; 2 Psychiatry, Cape Breton Regional Hospital, Sydney, Canada

## Abstract

**Introduction:**

Previous studies have shown that Peer Support Programs (PSPs) promote workforce wellness by supporting clinicians during times of heightened stress and vulnerability (Keyser, et al., 2021). Inclusion of case discussions in PSPs can provide opportunity for reflective practice, quality improvement, and professional development, in addition to strengthening clinicians’ resilience.

**Objectives:**

To describe the experience and perceived benefits reported by participants (psychiatrists) of a peer support and case discussion group meeting, of a clinical department of psychiatry (DOP) in Cape Breton, Canada, which the group calls “clinical café meeting”.

**Methods:**

Qualitative data collected, were informal comments (with focus on the participants’ experience and perceived benefits) from the group participants during the once a month, one-hour clinical cafe meetings.

**Results:**

From September 2015 to September 2021, attendance ranged from 2 to 10 participants. All participants voiced that, they see each meeting as an opportunity to “analyze their feelings and knowledge relevant to clinical practice situations, especially those associated with uncomfortable feelings (Atkins & Murphy reflective model, 1993), and challenges they face, in relation to the healthcare system. Many participants voiced how input from group participants help them with gaining a new perspective on practice situations that were discussed, and ideas on how they could deal with similar clinical situations or challenges, in a more robust way, in the future. Many participants also find the clinical café meetings to be helpful in consolidating their resilience.

**Conclusions:**

PSP (with case discussion) participants, in a Canadian DOP, described their experience of the group meetings, as beneficial, including contributing to strengthening of their resilience.

**Disclosure of Interest:**

None Declared

